# Characterization of Volatile Profile of Different Kiwifruits (*Actinidia chinensis* Planch) Varieties and Regions by Headspace-Gas Chromatography-Ion Mobility Spectrometry

**DOI:** 10.3390/foods15010152

**Published:** 2026-01-03

**Authors:** Lijuan Du, Yanan Bi, Jialiang Xiong, Xue Mu, Dacheng Zhai, Weixiang Chen, Hongcheng Liu, Yanping Ye

**Affiliations:** 1 Institute of Quality Standards & Testing Technique, Yunnan Academy of Agricultural Sciences, Kunming 650205, China; 36violet@163.com (L.D.); cm_biyn@163.com (Y.B.); 2 Dali Bai Autonomous Prefecture Inspection and Testing Institute, Dali 671000, China; cm_xiongjl@163.com; 3 Yunnan Jiahui Testing Technology Co., Ltd., Kunming 650200, China; cm_mux@163.com (X.M.); cm_zhaidc@163.com (D.Z.); cm_chenwx@163.com (W.C.)

**Keywords:** kiwifruit, VOCs, GC-IMS

## Abstract

The flavor and aroma of kiwifruit are largely influenced by the concentration of Volatile Organic Compounds (VOCs). To analyze the volatile profiles and identify characteristic aroma compounds, this study utilized Gas Chromatography-Ion Mobility Spectrometry (GC-IMS) to analyze the aromatic compounds sourced from seven major production regions in China and New Zealand, covering red-, green-, and yellow-fleshed varieties. A total of 77 VOCs were identified, with esters, aldehydes, and ketones as the dominant classes. Significant regional and varietal differences were observed: red-fleshed kiwifruits from Yunnan exhibited high levels of 2-Vinyl-5-methylfuran, Ethyl formate, and 1-Penten-3-one; green-fleshed fruits from Shaanxi were rich in Limonene and Methyl hexanoate, and those from Yunnan were rich in 1-Propanol and 1-Hexanol; and yellow-fleshed fruits from Henan were characterized by Methyl salicylate and 3-Hydroxy-2-butanone. Orthogonal partial least squares discriminant analysis (OPLS-DA) successfully classified kiwifruits by origin and variety, confirming the stability and predictive power of the model (Q2Y > 0.97). This study also elucidated the key metabolic pathways—including lipid oxidation, amino acid degradation, and terpenoid metabolism—underlying the formation of these characteristic VOCs. These findings provide a theoretical foundation for the biochemical regulation of kiwifruit flavor and support the development of origin-tracing and quality-assessment tools based on VOC fingerprints.

## 1. Introduction

Kiwifruit (*Actinidia chinensis* Planch), commonly referred to as “qiyiguo,” is a deciduous liana fruit tree belonging to the genus *Actinidia* within the family *Actinidiaceae*. This species demonstrates considerable adaptability, thriving in both temperate and subtropical climates [1]. The commercial value and consumer appeal of these varieties stem largely from their unique sensory attributes. For instance, red-fleshed kiwifruits are often characterized by their distinct tropical fruit aromas and high sugar-acid ratio; green-fleshed kiwifruits (e.g., ‘Hayward’) are widely recognized for their classic kiwifruit flavor and good storability, while yellow-fleshed kiwifruits (e.g., ‘Jinyan’) typically exhibit milder acidity and a citrus- and honey-like aroma profile (relevant literature citations have been added). These inherent inter-varietal differences provide an important biological context for investigating the diversity of their VOC profiles [2]. Globally, 54 species within the Actinidia genus have been identified, with 52 species natives to China [3]. Aroma characteristics are among the key determinants of the overall sensory quality of fruits. Extensive research has demonstrated a strong correlation between specific aroma profiles and consumer preference as well as purchase intent [4]. Therefore, elucidating the VOC characteristics of kiwifruits from different varieties and producing regions holds practical significance for understanding their market differentiation positioning and meeting consumer demands [5].

The VOCs present in kiwifruit are crucial in defining its flavor and aroma profile [4]. These compounds serve as vital quality indicators throughout the ripening process, exerting a significant impact on the fruit’s flavor and consumer acceptance. Research by Du, Lan, and colleagues has revealed that ‘Hayward’ kiwifruit contains elevated levels of green aroma compounds, such as 2-hexenal and hexanal, while ‘Hort16A’ kiwifruit is distinguished by a higher concentration of tropical aroma compounds, including ethyl butyrate, eucalyptol, and methyl mercaptan [5]. The primary VOCs in kiwifruit encompass esters, which impart a fruity aroma, alcohols, which contribute a grassy aroma, and ketones, which provide a floral aroma. The dynamic alterations in these compounds are modulated by various biotic and abiotic factors, notably the ethylene signaling pathway, temperature conditions, and diverse postharvest treatment methods [6]. Aroma components are intricately linked to commercial preservation and postharvest handling. Among environmental factors, storage temperature plays the most critical role in regulating aroma; inappropriate storage conditions can result in aroma degradation, adversely affecting not only volatile but also the nutritional and sensory quality of the fruit [4]. Cold storage at 5 °C facilitates fruit softening and the accumulation of sugars, such as sucrose and fructose; however, it inhibits the ethylene pathway, consequently suppressing the production of aroma volatile compounds [6,7]. During the post-ripening phase, there is an enhancement in the metabolism of carbohydrates, such as glucose, and amino acids, including valine and leucine. These metabolites serve as precursors for aroma volatile compounds, such as esters and aldehydes, indicating that the metabolism of sugars and amino acids may supply substrates necessary for aroma synthesis.

In current research on chemical components, while high-performance liquid chromatography (HPLC) and its ultra-high-performance variant (UPLC) are powerful tools for analyzing non-volatile or semi-volatile compounds, they are generally not directly used for the analysis of highly volatile aroma compounds without derivatization. In contrast, gas chromatography-mass spectrometry (GC-MS) is the gold standard for VOC analysis. Headspace-gas chromatography-ion mobility spectrometry (HS-GC-IMS), the technique employed in this study, as an emerging analytical method, exhibits unique advantages in the rapid fingerprinting analysis of VOCs without complex sample pretreatment [8]. GC-IMS (Gas Chromatography-ion mobility spectrometry) is a relatively new analytical technique that has emerged in recent years. It combines the high-resolution separation ability of GC with the high-sensitivity detection and rapid analysis characteristics of IMS. Compared with GC-MS, GC-IMS has several advantages, which can directly use head-space sampling or simple solid-phase micro-extraction (SPME) for sample introduction. GC-IMS also has high sensitivity. It can detect trace amounts of volatile components in the sample. For instance, in the identification of different varieties of fruits or the discrimination of different-origin agricultural products based on their volatile components, GC-IMS can more intuitively show the differences in volatile components between samples through the comparison of spectra, providing a more straightforward way for sample classification and quality evaluation [8,9]. Within the realm of food analysis, GC-IMS has achieved extensive application for various purposes, such as flavor and quality assessment, detection of trace toxic chemicals, and identification of adulteration. The technique is distinguished by several advantages, including high sensitivity, non-destructive analysis, rapid results, high reliability, ease of use, and cost-effectiveness [10]. For instance, GC-IMS has been utilized in flavor analysis to assess volatile organic compound production in fruits such as pomegranate [11], passion fruit [12], and peach [13]. Additionally, it has been applied in comparative analyses to identify unknown flavors in various meats, including donkey meat and shrimp (crayfish) meat [7]. This study utilizes GC-IMS to investigate the internal quality and aroma profiles of kiwifruits with different flesh colors from several key production regions. The primary objective is to establish a volatile compound fingerprint to identify characteristic substances. The specific objectives are: (1) to elucidate the aroma composition of kiwifruits from major production regions in China and compare them with imported varieties; (2) to conduct a comparative analysis of the aroma chemical components of kiwifruits with different flesh colors from diverse major production regions; and (3) to classify the aroma chemical components of kiwifruits using orthogonal partial least squares discriminant analysis (OPLS-DA), and to explore potential correlations between quality indicators, VOCs, and GC-IMS data. The methodological framework established in this study, which combines HS-GC-IMS with multivariate statistical analysis, can effectively distinguish kiwifruits from different varieties and producing regions, as well as identify their key characteristic aroma compounds. These findings provide potential technical tools and chemical evidence for kiwifruit quality evaluation and geographical origin traceability, and lay the groundwork for preliminary research on the material basis of targeted flavor quality improvement through postharvest management strategies in the future.

## 2. Materials and Methods

### 2.1. Sample Collection and Preparation

Kiwifruits that had attained commercial maturity were procured from producers and fresh fruit markets. A total of 93 samples were acquired, originating from major kiwifruit-producing regions across 5 distinct provinces in China (Yunnan, Shaanxi, Sichuan, Guizhou, Chongqing, Henan, and Hunan) as well as New Zealand. All specimens harvested from August to October 2024, including samples from kiwifruit production bases in major kiwifruit-producing provinces across China (such as Shaanxi, Sichuan, Guizhou, and Yunnan) as well as New Zealand. During the commercial maturity stage of kiwifruits, sampling was conducted by taking one sample of the same variety from each production base (with random sampling applied). Each sample weighed 5 kg, which was properly packaged and transported back to the laboratory on the same day under refrigeration. When the fruit firmness dropped between 3.9 and 9.8 N/cm^2^, 10 fruits were randomly selected from each sample for phenotypic trait determination. For the remaining part of the samples: one portion was homogenized, quick-frozen, and stored at −18 °C for volatile organic compounds (VOCs) determination. Specimens exhibiting uniform size and shape, and devoid of mechanical damage (such as dents and scratches), cracks, or signs of decay, were selected for further processing. Fruit firmness was measured using a durometer (GYJ-4, Gongyouji Industrial Technology (Shenzhen) Co., Ltd. (Shenzhen, China)). The test results, initially expressed in kg/cm^2^, were converted by multiplying with 9.8 N/kg, and the final was presented in N/cm^2^. Each sample was analyzed in three biological replicates. Detailed sample information, including origin, species, and flesh color, is presented in Table 1 and Table S1.

### 2.2. Volatile Extraction and Analyses by GC-IMS

A 3.00 g sample of kiwifruit was placed into a 20 mL headspace extraction vial. It was incubated in a shaking incubator at 45 °C with a rotation speed of 500 r/min for 15 min. Then, 0.5 mL of the headspace sample was injected into the auto-sampler in splitless mode using a 1 mL gas-tight heated syringe. The injection temperature was set at 80 °C. The analysis was conducted using FlavourSpec^®^ gas chromatography-ion mobility spectrometry (G.A.S. GmbH, Dortmund, Germany), with a CTC-PAL 3 static headspace auto-sampler. Qualitative analysis of substances is conducted using VOCal software (0.4.03) and the NIST database and IMS database (G.A.S. GmbH, Dortmund, Germany).

### 2.3. Qualitative Analyses of Volatiles

GC-IMS was conducted using an MXT-WAX column (30 m, 0.53 mm ID, 1.0 μm df, RESTEK, Bellefonte, PA, USA) at 60 °C, with IMS at 45 °C. Nitrogen served as the carrier and drift gas. Samples were incubated at 45 °C with a speed of 500 rpm for 15 min, and 500 μL was injected. The GC temperature program was as follows: 0–2 min at E1 = 75 mL/min, E2 = 2 mL/min; 2–10 min at E1 = 75 mL/min, E2 = 10 mL/min; 20–30 min at E1 = 75 mL/min, E2 = 100 mL/min, with an operating voltage of 500 V (corresponding to an electric field strength of approximately 400 V/cm) (E1 refers to the carrier gas (N_2_) flow rate, and E2 refers to the drift gas flow rate.). A mixed standard was used to establish a calibration curve for retention time and retention index. Subsequently, the retention index of the target analyte was calculated from its retention time. Qualitative analysis of the target analyte was performed by retrieving and matching the obtained data against the built-in GC retention index database NIST and IMS migration time database in the VOCal data processing software. Quantitative analysis of the volatile substances was carried out using 2-nonanone as the internal standard [14]. All measurements were performed in triplicate, and the results were reported as mean values with relative standard deviation (RSD).

### 2.4. Statistical Analyses

In this study, graphical analyses were conducted using ORIGIN and SIMCA software. For data exhibiting uniform variance, a one-way analysis of variance (ANOVA) was performed, followed by Holm-Bonferroni multiple comparisons and significance testing (*p* < 0.05). OPLS-DA analysis was conducted using SIMCA, with simultaneous calculation of VIP values and performance of permutation tests. The test was performed with 200 permutations, and the intercepts of the regression lines of the explained variance (R^2^Y) and predictive ability (Q^2^) of the permuted models at the origin were calculated to evaluate the significance of the model and avoid overfitting. Specifically, training set (70%) was used for model construction and internal validation (including the permutation test), and independent test set (30%) was used solely for a one-time, final external evaluation of predictive performance after the model was finalized, providing an unbiased estimate of the model’s true predictive capability. Two-dimensional spectra and fingerprint spectra of volatile components were generated using the Reporter and Gallery Plot plugins in the VOCal data processing software, respectively. To explore potential signaling pathways influencing differential metabolites, significantly differential volatile compounds with a VIP value > 1.0 were used as the input list, and pathway enrichment analysis was performed using the MetaboAnalyst 5.0 platform (https://www.metaboanalyst.ca (accessed on 30 June 2025). Compound identifiers were mapped to the KEGG database via their chemical names or CAS numbers. The hypergeometric distribution test was adopted for enrichment analysis, with pathway significance thresholds set at *p*-value < 0.05 and false discovery rate (FDR) < 0.1.

## 3. Results and Discussion

### 3.1. Volatile Profiling Analysis of Kiwifruits

The three varieties of kiwifruit demonstrated distinct aroma profiles, characterized by variations in both the type and concentration of volatile compounds. The major objective of this research was to depict and compare the (VOC profiles of fruits at commercial harvest stages from different varieties and their typical producing regions at a “current status” level, which reflects the characteristics of products available to consumers. The primary chemical classes identified were esters, aldehydes, and ketones. Esters contributed to the sweet aroma of the kiwifruit, while aldehydes and ketones were associated with fresh and herbaceous aromas. Through a comprehensive analysis of the aroma compounds present in these kiwifruit varieties, which included database matching and retention index (RI) comparison, a total of 77 compounds were identified (Table S1). The composition of VOCs was as follows: Esters (23 compounds) constituted between 24.8% and 35.1% of the total VOCs; Aldehydes (17 compounds) accounted for 15.4% to 28.4%; Ketones (10 compounds) comprised 10.6% to 18.2%; Alcohols (9 compounds) ranged from 8.02% to 14.3%; Furans (5 compounds) made up 4.99% to 9.93%; Terpenoids (5 compounds) represented 3.35% to 10.9%; Hydrocarbons (5 compounds) constituted 2.97% to 9.10%; and Ethers (3 compounds) accounted for 1.03% to 13.3% of the total VOCs.

**Figure 1:**
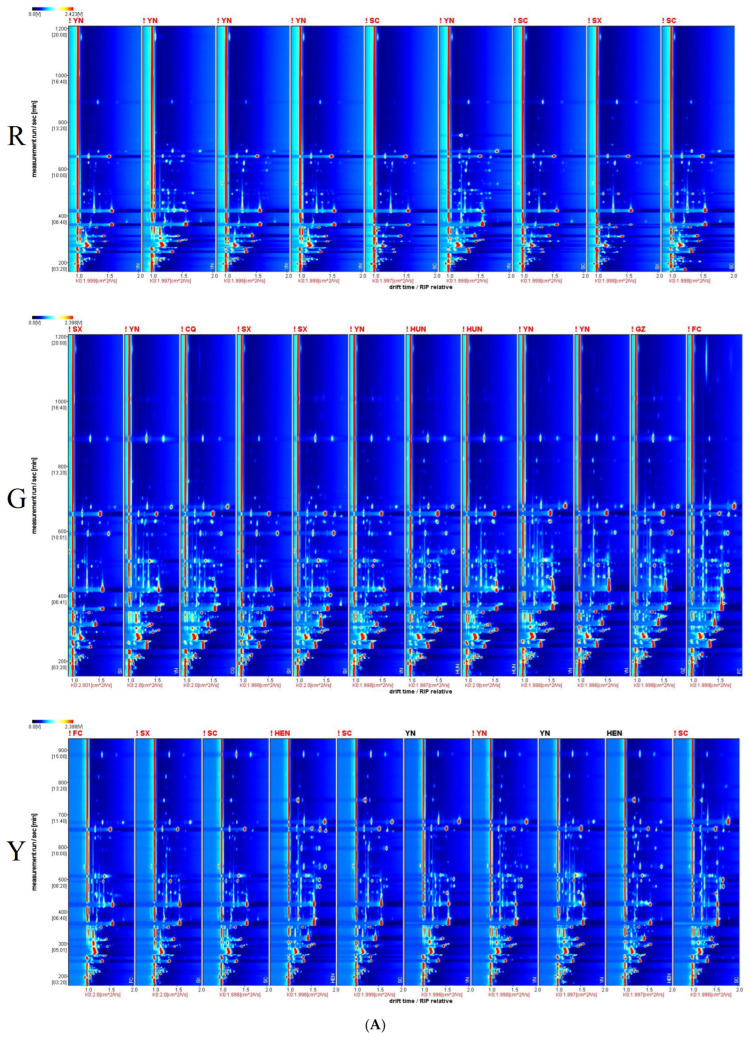

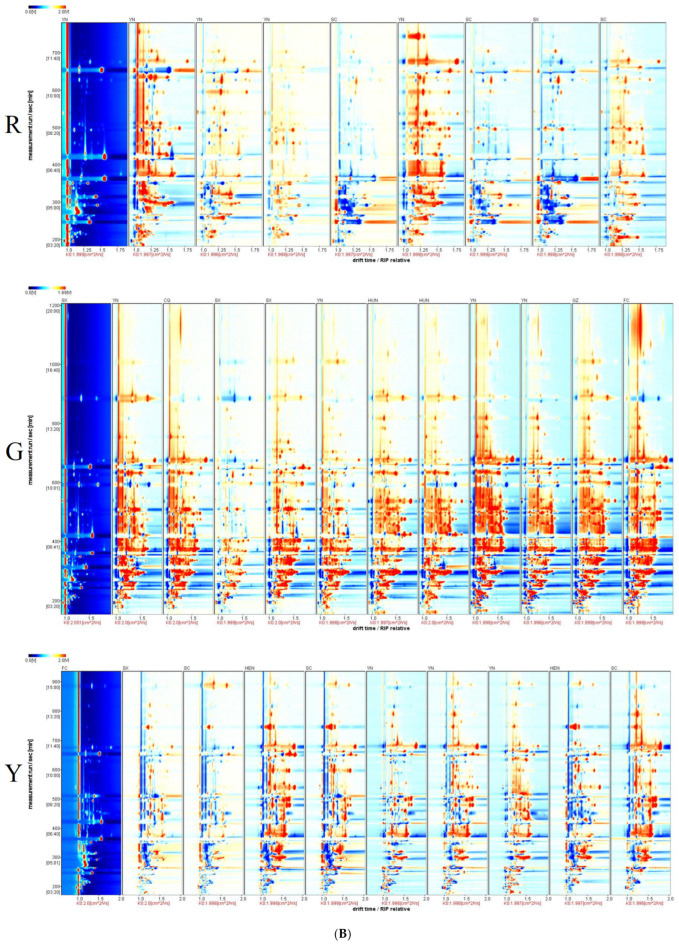

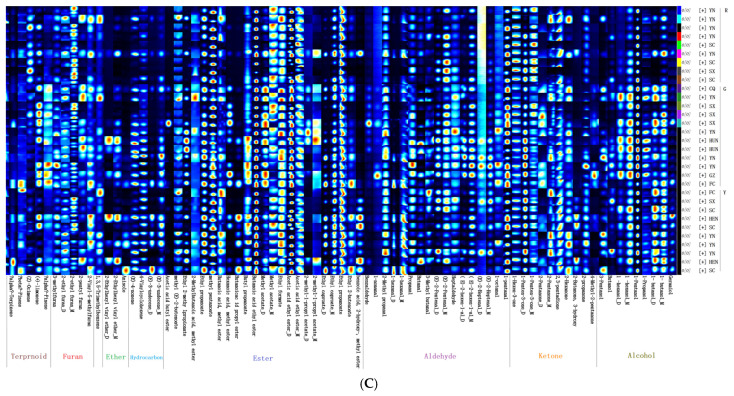
GC-IMS spectrum diagram of kiwifruits. Notes: (**A**): the vertical view of 2D-topographic plots: (1) the entire background of the figure is blue, and the red vertical line at the abscissa of 1.0 represents the RIP peak (Reaction Ion Peak, normalized); (2) the ordinate represents the retention time (s) of gas chromatography, while the abscissa represents the ion migration time (normalized); (3) each dot on both sides of the RIP peak corresponds to one volatile organic compound; (4) the color indicates the concentration of the substance, among which white denotes low concentration, red denotes high concentration, and darker colors represent higher concentrations. (**B**): a differential comparison mode: (1) the spectrum of one sample is selected as the reference, and the spectra of other samples are subtracted from the reference; (2) if the VOC profiles of two samples are identical, the background after subtraction will be white; red indicates that the concentration of the substance is higher than that in the reference, and blue indicates that the concentration is lower than that in the reference; (3) YN-produced was used as the reference for red-fleshed kiwifruit samples, SX-produced for green-fleshed kiwifruit samples, and FC-produced for yellow-fleshed kiwifruit samples; (**C**): the Gallery Plot of VOCs: (1) each row in the figure represents all signal peaks selected from one tobacco leaf sample; (2) each column represents the signal peak of the same volatile organic compound across different tobacco leaf samples; (3) the figure provides comprehensive VOC information for each sample and clearly illustrates the VOC differences between samples; (4) the letters M and D in parentheses after the substance names represent the monomer and dimer forms of the compound, respectively.

Figure 1 presents the GC-IMS fingerprint spectra of three kiwifruit varieties. Following background subtraction using the first sample as a reference, a comparative analysis revealed distinct aroma profiles among the varieties. The red-fleshed kiwifruit from Yunnan exhibited a relatively higher volatile content, while the green-fleshed varieties showed peak concentrations in FC samples. In contrast, the yellow-fleshed types from Henan demonstrated superior the total relative signal intensity. Notable distribution patterns were observed: the red-fleshed varieties were predominantly characterized by aldehydes and esters; the green-fleshed types displayed a broad distribution across alcohols, aldehydes, esters, ketones, terpenoids, and furans; and the yellow-fleshed cultivars exhibited similar chemical diversity (quantitative comparison results based on the Shannon-Wiener index) but at lower concentrations compared to their green-fleshed counterparts. Key differential compounds were identified in the green-fleshed varieties, which contained elevated levels of ethyl propanoate, 2,3-pentanedione, ethyl caproate, 2-pentanone, butanoic acid ethyl ester, butanal, and acetic acid butyl ester. These compounds contributed characteristic fruity, sweet, and grassy, complemented by distinctive nutty, buttery, and rum-like nuances. Yellow-fleshed kiwifruit exhibited a higher relative abundance of Methyl salicylate (benzoic acid, 2-hydroxy-, methyl ester), 3-Hydroxy-2-butanone, Propyl butyrate, 2-Ethylhexyl vinyl ether, (E)-3-Undecene, and Anisole, which imparted subtle grassy, oily, and ice cream-like aromas.

Ethyl propanoate and Ethyl caproate constitutes the primary volatile esters present in kiwifruit, thereby classifying them as core aroma compounds. The kiwifruit varieties exhibiting the greatest abundance of esters include the red-fleshed variety from Yunnan, the green-fleshed variety from Hunan, and the yellow-fleshed variety from Sichuan. Esters are responsible for imparting complex fruity to kiwifruit. Notably, kiwifruit from Chinese production regions exhibit a more abundant and diverse ester profile [15].

(E)-2-heptenal and 1-Hexanal were present in the highest concentrations and played a significant role in shaping the aroma profile of kiwifruit, since such compounds are often described in the literature as contributing ‘green grass’ and ‘green leaf’ aromas (references provided), the results of our analysis may be related to the formation of these aroma attributes. The highest aldehyde content was observed in kiwifruit cultivated in Yunnan across all three varieties (red-, green-, and yellow-fleshed). According to the reported literature, aldehydes predominantly contribute grassy and vegetal, suggesting that Yunnan kiwifruit possess more pronounced fresh-green aromas [15].

The major ketones identified included 3-hydroxy-2-butanone, 2,3-pentanedione, 2-pentanone, and 4-methyl-2-pentanone. These compounds significantly influenced the aroma of kiwifruit and are considered essential aroma components. Varieties rich in ketones included red- and green-fleshed kiwifruit from Yunnan, as well as yellow-fleshed cultivars from Hunan and Sichuan. Ketones impart fruity, floral, creamy, and woody nuances to the aroma profile. The increased diversity of ketones in kiwifruit cultivated in China, as opposed to those from New Zealand, indicates a more intricate and nuanced aromatic profile in the domestically produced fruit [15].

Among the alcohols, 1-butanol and 1-propanol were identified as significant volatile compounds. Similarly, the three ether compounds detected played a substantial role in influencing the aroma, qualifying as essential components. In the category of furans, 3-methylfuran and 2-pentylfuran were predominant, imparting roasted and earthy. The terpenoid alpha-terpinene was the primary contributor, providing herbal and citrus-like. For hydrocarbons, (E)-3-undecene was the main compound, contributing waxy and green undertones. Collectively, these compounds enhanced the aromatic complexity, particularly in kiwifruit grown in Yunnan, introducing descriptive scope covers various aroma characteristics such as fruity, green, mellow, and roasted aromas as reported in the literature [15].

### 3.2. Multivariate Analysis of Kiwifruits

The OPLS-DA model, as a supervised learning algorithm, seeks to elucidate the relationship between metabolites and sample categories through partial least squares regression. This approach enhances the differentiation between sample groups, facilitates the identification of inter-group variations, and aids in recognizing characteristic variables specific to each group. The parameters R2Y and Q2Y denote the explained variance and predictive capability within randomized Y-variable models, respectively. Values approaching unity for both parameters suggest stronger explanatory power, improved classification performance, and superior predictive ability of the model. A Q2Y value exceeding 0.5 indicates an effective model, whereas a Q2Y value greater than 0.9 denotes an excellent model. To rigorously evaluate model performance, we explicitly report results from both the training and the independent test sets. During model construction and 7-fold cross-validation on the training set (70% of samples), the model demonstrated high explanatory and predictive capability. More critically, when applied to the held-out test set (30% of samples), which was not involved in any stage of model building, the model maintained robust predictive performance.

In the model, variables with Variable Importance in Projection (VIP) values exceeding 1 were identified as key discriminatory compounds for differentiating kiwifruit samples based on their geographical origins. A total of 35 aroma compounds were selected for red-fleshed kiwifruit, 31 for green-fleshed, and 28 for yellow-fleshed varieties. Variations in the concentrations of these compounds offer comprehensive insights into the overall aromatic profile, encompassing its foundation, richness, and complexity. Collectively, these compounds emit a diverse array of aromatic, including floral, fruity, sweet, green, nutty, chocolate-like, alcoholic, roasted, cereal, and creamy characteristics, in summary, these compounds collectively constitute the characteristic chemical fingerprints that distinguish kiwifruits of different varieties and producing regions. These substances may serve as characteristic markers for distinguishing the geographical origins of the three kiwifruit types.

**Figure 2:**
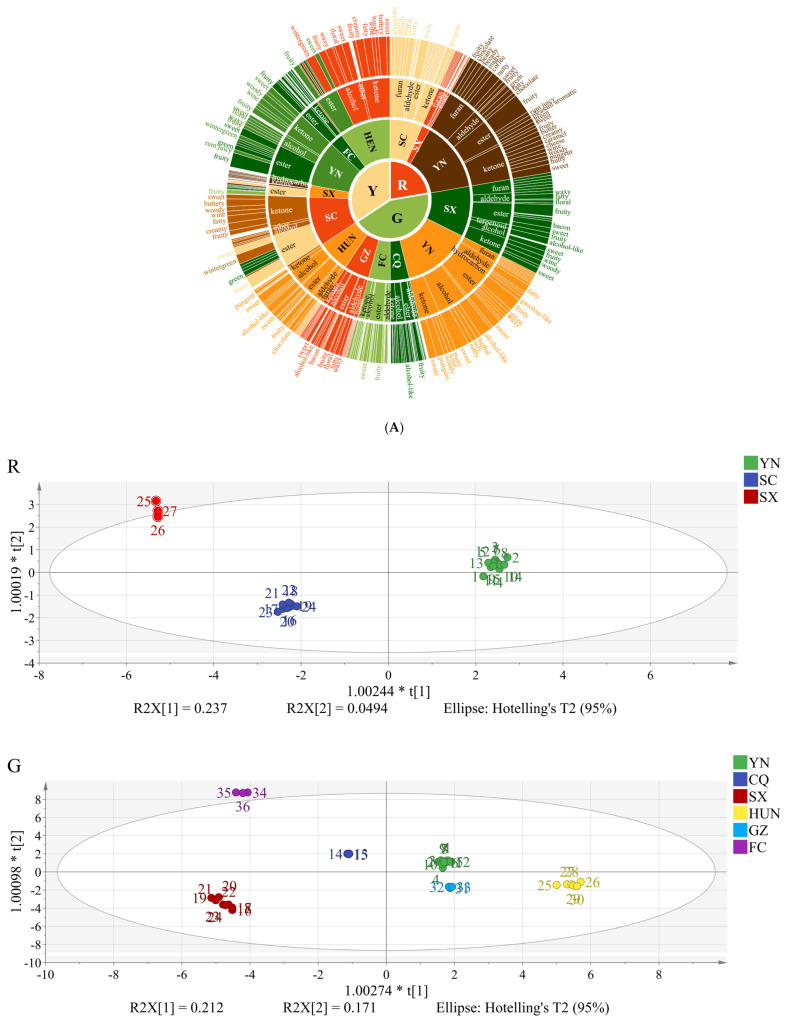

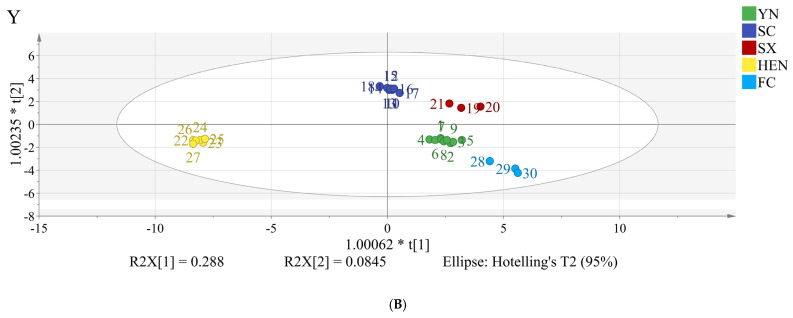
VOCs and classifications of kiwifruits in different varieties. Notes: (**A**), flavor wheel of kiwifruit different varieties. (**B**), OPLS-DA scores of different varieties from different regions.

As depicted in Figure 2A, the aromatic profiles of red-fleshed kiwifruit are primarily composed of ketones, esters, hydrocarbons, and furans, with relative concentrations ranked as follows: Yunnan > Sichuan > Shaanxi. In contrast, green-fleshed varieties exhibit greater aromatic complexity, encompassing ketones, esters, aldehydes, alcohols, hydrocarbons, terpenoids, and furans, with concentration gradients observed as Yunnan > Shaanxi > Hunan > Guizhou > Chongqing, where domestic samples consistently surpass imported ones. Yellow-fleshed kiwifruit is characterized by predominant concentrations of ketones, esters, hydrocarbons, ethers, and alcohols, arranged in the order of Sichuan > Henan > Yunnan > Shaanxi, with domestic samples again demonstrating superior aromatic intensity compared to their foreign counterparts. As depicted in Figure 2B, the OPLS-DA model effectively identified and distinguished the geographical origins of all three kiwifruit varieties. The cumulative explanatory parameters, R2Y, were 0.990 for red, 0.992 for green, and 0.992 for yellow kiwifruits, while the predictive parameters, Q2Y, were 0.972 for red, 0.983 for green, and 0.982 for yellow, respectively on the training set. The model exhibited excellent fit and predictive robustness. The corresponding root mean square errors were low for both estimation (RMSEE were 0.0434695 for red, 0.0503641 for green and 0.0338597 for yellow) and cross-validation (RMSECV were 0.0593211 for red, 0.070779 for green and 0.0417436 for yellow). The proximity of RMSEE and RMSECV values indicates that the model possesses strong generalization capability without overfitting to the training data. External validation on the independent test set demonstrated that the prediction accuracies for red-, green-, and yellow-fleshed kiwifruits all reached 100%. This outstanding prediction accuracy further corroborates the reliability of the aforementioned internal validation results. The significant differences in VOCs among kiwifruits from different producing areas, successfully captured and predicted by our OPLS-DA model on both training and unseen test data, underscore the model’s practical utility. The analytical framework provides a reliable and validated technical tool for distinguishing kiwifruits of specific varieties and origins involved in this research (Table S2).

**Figure 3:**
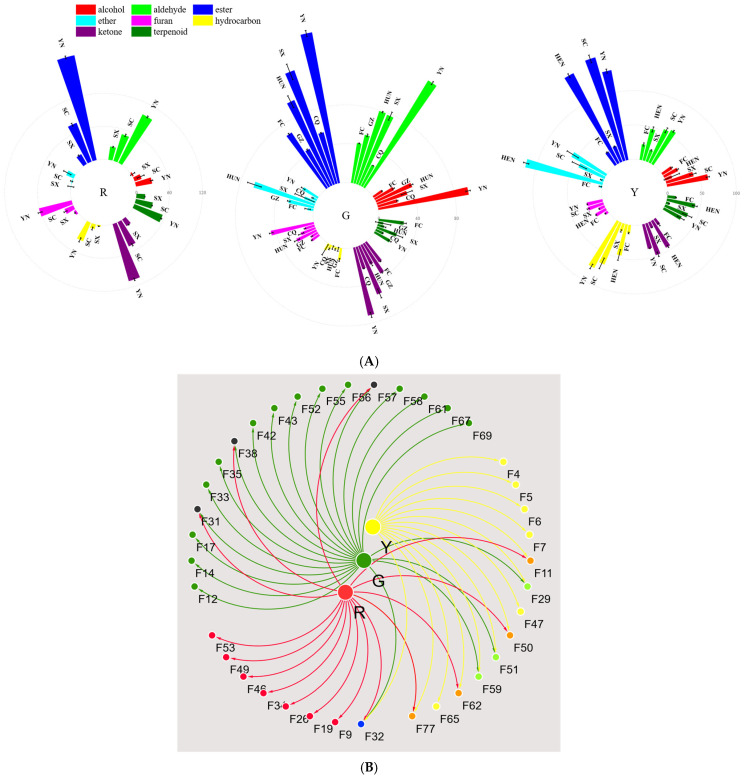

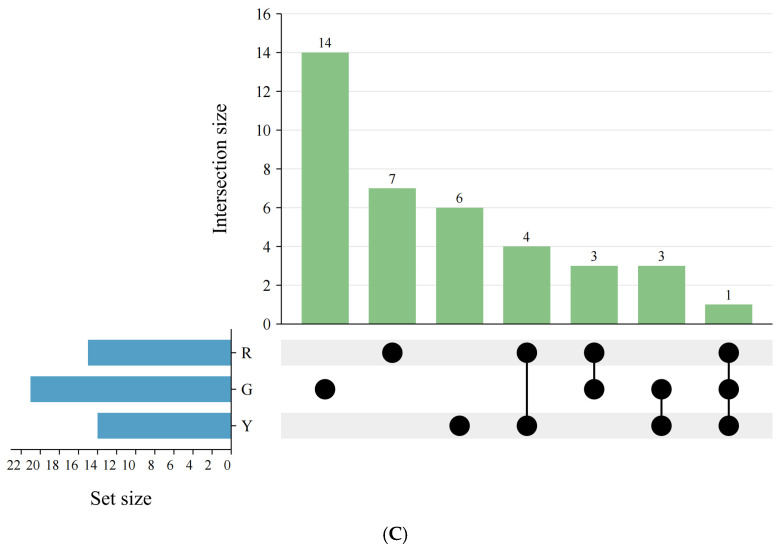
VOCs in different varieties and regions. Notes: (**A**) means the radial bar of different varieties, respectively. (**B**,**C**) are the network and Venn plot. The specific details of Figure (**B**) were as follows: the red, green, and yellow lines/dots in the figure represent the unique characteristic aroma components of red-fleshed, green-fleshed, and yellow-fleshed kiwifruits respectively; the brown, orange, and light green dots denote the aroma components shared by two kiwifruit types, namely red-fleshed & green-fleshed, red-fleshed & yellow-fleshed, and yellow-fleshed & green-fleshed kiwifruits in sequence; the blue dots indicate the aroma components common to all three types.

Among the various types of kiwifruits, green-fleshed varieties exhibit the most diverse flavor profile, whereas yellow-fleshed varieties display greater uniformity in their volatile composition. Notably, kiwifruits cultivated in Yunnan consistently exhibit higher concentrations of key flavor compounds. An analysis of differential VOCs with a VIP score greater than 1 and a *p*-value less than 0.05 indicated that esters comprised 30–35% of the total volatile compounds across the three kiwifruit varieties. These esters predominantly originate from acid-alcohol condensation reactions, with short-chain esters being the primary contributors to the characteristic fruity-sweet aroma profile. Notably, the ester content was highest in red-fleshed varieties, followed by green-fleshed and yellow-fleshed varieties. Aldehydes comprised 12.3% to 22.9% of the total volatile compounds, primarily originating from lipid oxidation and degradation processes [12,16]. These compounds significantly contributed to the characteristic fresh-green, malty, and fruity aroma profiles of kiwifruit, with concentration levels observed in the order of green-fleshed > red-fleshed > yellow-fleshed varieties. Ketones constituted 9.2% to 20.0% of the total volatile compounds, primarily arising from carotenoid degradation [17], fatty acid oxidation [17], and terpenoid metabolism [18]. These compounds were principally responsible for the characteristic fruity-sweet, baked-sweet, and creamy aroma profiles in kiwifruit, with their concentrations following the varietal distribution of red-fleshed > green-fleshed > yellow-fleshed varieties. Ethers accounted for 2.0% to 13.7% of the total volatile composition, primarily originating from terpenoid biosynthesis and oxidative reactions [18]. These compounds were instrumental in defining the characteristic clean-sweet, baked, and creamy aroma profiles of kiwifruit, with concentration levels following a varietal gradient: yellow-fleshed > green-fleshed > red-fleshed cultivars. Alcohols constituted 5.3% to 12.6% of the total volatile profile, mainly produced through lipid oxidation [12], amino acid degradation [19], and microbial fermentation [16]. These compounds significantly contributed to the fruity-sweet, floral, and creamy aroma characteristics of kiwifruit. However, elevated alcohol concentrations may result in undesirable pungent or putrid odors or solvent-like off-flavors. The distribution of alcohol content exhibited a distinct varietal pattern: green-fleshed > yellow-fleshed > red-fleshed cultivars (Figure 3).

### 3.3. Unique Aroma Compounds of Kiwifruits

**Figure 4:**
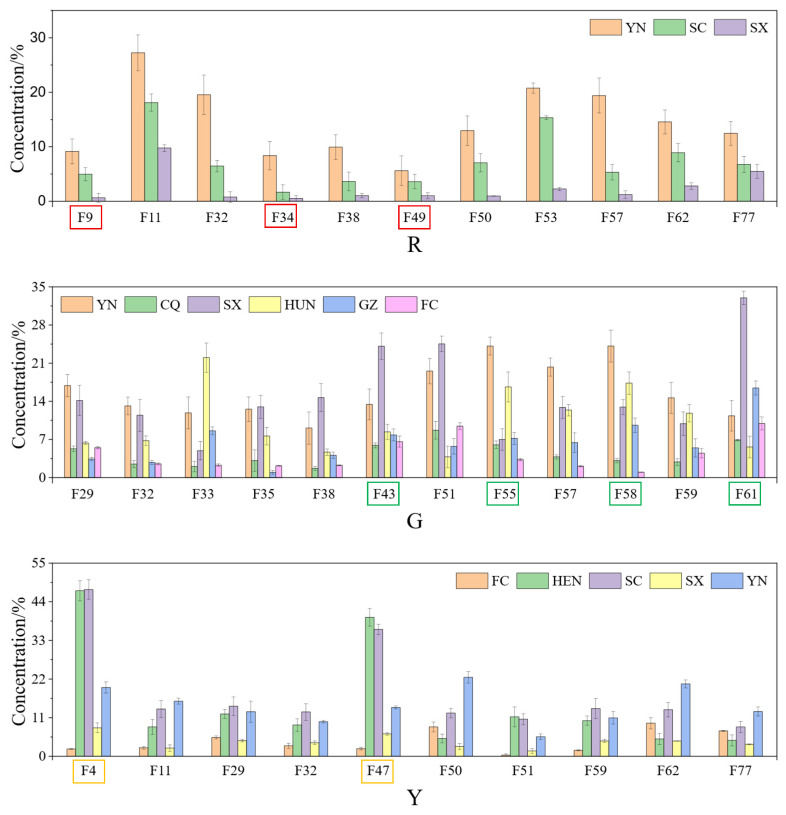
Common and unique aroma compounds of Kiwifruits.

The enrichment of specific components in kiwifruits varies across different varieties and regions of production (Figure 4). The R variety is distinguished by the presence of characteristic aromatic compounds, including 2-Vinyl-5-methylfuran, Ethyl formate, and 1-Penten-3-one, which collectively contribute to its sweet and fruity fragrance profile, particularly in kiwifruits cultivated in the YN region, imparting berry-like [20] and caramelized woody undertones [21]. ① 2-Vinyl-5-methylfuran is detected at low levels in fresh fruits, contributing less than 1% to the volatile compound composition This compound predominantly originates from the Maillard reaction during storage. ② Ethyl formate, or other alcohols and esters, via the action of enzymes such as alcohol dehydrogenase (ADH) and alcohol acyltransferase (AAT) produces aldehyde precursors, which generates the oxidation of polyunsaturated fatty acids through lipoxygenase (LOX) pathway [16]. As a compound with dual characteristics, it emits a pleasant aromatic odor when present in appropriate concentrations. ③ 1-Penten-3-one exhibits a distinctive “fresh spiciness” aroma [22]. This compound often coexists with other volatile constituents, such as aldehydes, alcohols, and esters, collectively contributing to the overall aroma profile of fruits. In the present study, the relative concentrations of 2-Vinyl-5-methylfuran, Ethyl formate, and 1-Penten-3-one in samples from YN were relatively high, at 1.45%, 1.55%, and 2.24%, respectively, whereas those in samples from SX were comparatively low, at 0.62%, 1.05%, and 1.25%, respectively. The underlying reason may be that the lower average temperature and larger diurnal temperature variation affect plant enzyme activity, thereby altering the pathways of lipid oxidation and volatile compound synthesis to produce 2-vinyl-5-methylfuran, ethyl formate, and 1-penten-3-one [16,23].

The characteristic aromas of the G variety, distinguished by a unique grassy scent, encompass compounds such as Limonene, 1-Propanol, 1-Hexanol, and Methyl hexanoate, particularly in samples produced in SX, and they play a significant role in imparting a distinct sweet and fruity aroma [24]. ① Limonene, the primary volatile terpenoid compound found in fruits, is characterized by its high volatility and low solubility in water. The differences in limonene content observed in this study may reflect background differences in fruit metabolism, and the specific causes (genetic, environmental, or postharvest factors) require further research [25]. ② 1-Propanol primarily originates from the activation of the LOX pathway and the increased activity of decarboxylases involved in the degradation of amino acids, particularly valine and isoleucine. It may serve as an indicator of anaerobic metabolism during the deterioration of fresh fruits in storage [16]. Consequently, 1-propanol acts as a double-edged sword; it contributes a desirable grassy aroma when present at appropriate levels. ③ 1-Hexanol is a significant C6 volatile compound found in fruits, serving as a primary contributor to the “green note” aroma. It is synthesized from fatty acids, such as linolenic and linoleic acids, through catalytic oxidation mediated by LOX enzymes. The presence and concentration of 1-Hexanol are critical in determining the “grassy” or “herbaceous” aroma profile of fruits [26]. ④ Methyl hexanoate, a characteristic aroma compound in fruits, is catalyzed by pectin methyltransferases [27]. In kiwifruit, the enzymatic pathways of LOX and AAT constitute fundamental components of the biosynthetic network responsible for flavor compound production. LOX, which functions as a crucial enzyme in the generation of volatile aldehydes and alcohols, facilitates the oxidative cleavage of fatty acids, resulting in the formation of volatile aldehydes and alcohols. Subsequently, the AAT-members of the BAHD acyltransferase superfamily, are essential enzymes catalyzing the synthesis of volatile esters—esterifies these alcohols, producing compounds that significantly contribute to the fruit’s aroma [28,29]. LOX facilitates the conversion of linoleic and linolenic acids into their respective hydroperoxides, thereby catalyzing the oxidation of polyunsaturated fatty acids (PUFAs) to produce fatty acid hydroperoxides. Subsequently, under the influence of hydroperoxide lyase (HPL), these hydroperoxides are transformed into volatile aldehydes, such as hexanal and hexenal. These aldehydes are then reduced to their corresponding alcohols, including hexanol and hexenol, through the action of alcohol dehydrogenase (ADH) [30,31]. AATs further catalyze the esterification reaction between these alcohols, derived from the LOX pathway, and acetyl-CoA, resulting in the formation of volatile esters [30]. Various AATs demonstrate specific affinities for alcohol and acyl-CoA substrates, resulting in the synthesis of diverse ester compounds, such as hexyl acetate and (Z)-3-hexenyl acetate [30]. Consequently, the alcohol precursors produced via the LOX pathway are crucial substrates for ester formation through the AAT pathway [29]. So, the relative concentration of Limonene was notably high in green-fleshed kiwifruits cultivated in the SX region, accounting for 2.68%. Conversely, the relative concentrations of 1-Propanol and 1-Hexanol were significantly elevated in green-fleshed kiwifruits from the YN region, with values of 2.77% and 3.20%, respectively. In contrast, the relative concentrations of these three VOCs were markedly lower in kiwifruits from the CQ production area, with percentages of 1.12%, 0.78%, and 1.04%, respectively. Methyl hexanoate exhibited distinct regional characteristics, showing a high relative concentration in kiwifruits from the SX region (5.48%) and a low relative concentration in those from the HUN region 0.93%).

Methyl salicylate, and 3-Hydroxy-2-Butanone (Acetoin) collectively define the characteristic aromatic profile of the Y variety, contributing to its distinctive rose-like fragrance, especially in samples produced in HEN, where they impart a refreshing herbal aroma complemented by warm, creamy undertones [32]. ① MeSA imparts a distinct ‘wintergreen’ or ‘mint-like’ scent and affects aroma profiles by modulating fatty acid metabolic pathways. ② Acetoin provides fruity, sweet, or milky scent characteristics. It works synergistically with diacetyl, acetaldehyde, and other substances to boost aroma intensity, significantly contributing to fruit [33]. As an aroma precursor or end product, it often acts synergistically with diacetyl to enhance the perception of creamy, fruity, or sweet in fruits [33]. In this study, the relative concentrations of Methyl salicylate and Acetoin were notably higher in samples produced in HEN, measuring 7.86% and 6.59%, respectively, compared to those produced in SX, which exhibited lower concentrations of 2.18% and 1.55%, respectively. The underlying reason might be that the different temperature fluctuations influence the activity of methyl salicylate synthase (SAMT), butanedione reductase, and Acetoin-producing enzymes, such as LOX, and further affect the synthesis of MeSA and Acetoin [34].

The biosynthesis of VOCs is dependent on the catalytic activity of specific enzymes that demonstrate significant temperature sensitivity, including LOX, ADH, and AAT. Optimal temperature conditions expedite the fruit ripening process, allowing ethylene-induced esters to attain maximal concentrations in a reduced period [25]. Additionally, with increasing altitude, air temperature declines and the diurnal temperature range broaden, which may facilitate the accumulation of sugars and flavor compounds. At elevated altitudes, both light intensity and ultraviolet (UV) radiation levels are elevated, may promoting the synthesis of protective secondary metabolites, including phenols and flavonoids. Variations in altitude also might lead to changes in soil types, drainage conditions, and precipitation patterns, which subsequently impact plant root development, water absorption, and nutrient utilization. These factors may have contributed to the synthesis of VOCs [35]. Furthermore, the microclimate data (e.g., temperature, humidity at sampling sites) were not measured, and that this represents an important avenue for future research to confirm the mechanisms behind the observed patterns. The variety- and origin-specific VOC fingerprint database established provides a basic reference framework and analytical method for the long-term monitoring and assessment of the stability and evolutionary trends of kiwifruit flavor quality under dynamic climatic conditions in the future.

### 3.4. Pathway of Common and Unique Aroma Compounds

The formation of characteristic VOCs in kiwifruits from various production areas and cultivars is governed by distinct metabolic pathways. The unique flavor profiles of kiwifruits cultivated at different origins are influenced by metabolic rate, enzyme activity, and ripening process, and further exacerbate the impact on VOCs [23]. The primary biochemical pathways that regulate alterations in aroma components during fruit ripening and storage encompass fermentative metabolism, lipid oxidation, amino acid degradation, and terpenoid metabolism [16].

Fermentative Metabolism: Hypoxic conditions can induce fermentative metabolism. This process involves the catalytic action of pyruvate decarboxylase (PDC) and ADH which facilitate the production of ethanol, 1-propanol, and 1-butanol, among other compounds, resulting in an off-odor characterized by a “wine-like taste” [16,36]. Ester compounds, including ethyl formate and ethyl propanoate, are synthesized through esterification reactions between alcohols and acyl-CoA, catalyzed by AATs [37,38]. Additionally, ketone compounds such as acetoin (3-hydroxy-2-butanone) are derived from pyruvate via the action of α-acetolactate decarboxylase [39,40].

Amino Acid Degradation: Amino acids undergo transamination and decarboxylation processes, leading to the formation of volatile compounds. Furan derivatives, including 2-vinyl-5-methylfuran and 2-ethylfuran, are produced through the thermal degradation of sugars or amino acids and contribute to a “caramel-like flavor.” Benzene ring derivatives, such as methyl salicylate, which originate from phenylalanine metabolism, impart a herbal aroma. Additionally, branched-chain esters and enones, such as methyl (E)-2-butenoate and 1-penten-3-one, are formed from the degradation of branched-chain amino acids, such as isoleucine.

Terpenoid metabolism involves the synthesis of limonene through either the mevalonate (MVA) or the methylerythritol phosphate (MEP) pathways, culminating in the cyclization of geranyl pyrophosphate (GPP) [41,42].

Lipid oxidation involves the generation of C6-C9 aldehydes and ketones, including compounds such as 1-Hexanal, 1-Octanal, and Butanal, through the β-oxidation of linoleic and linolenic acids, as well as the LOX pathway. In enzymatic reactions, LOX catalyzes the formation of hydroperoxides, which are subsequently converted into aldehydes by hydroperoxide lyase (HPL) [43]. For example, aldehydes like 3-Methyl butanal are produced via the Strecker degradation of leucine, wherein leucine is initially transformed into α-ketoisocaproic acid by transaminase, followed by its conversion to isovaleraldehyde by decarboxylase, and ultimately oxidized to form 3-Methyl butanal [43,44]. In addition, alcohols react with acyl-CoA in the presence of AATs to form esters. Short-chain esters, such as acetic acid ethyl ester, ethyl formate, and ethyl propanoate, are known to impart a sweet aroma to fruits, while long-chain esters, including butanoic acid methyl ester and hexanoic acid methyl ester, contribute to fruity aromas. Further, under conditions of mechanical damage, low-temperature stress, or enzymatic reactions in fruits, unsaturated fatty acids (UFAs) in membrane lipids produce C6-C9 aldehydes and ketones via the LOX pathway [45]. Straight-chain aldehydes, such as 1-hexanal, 1-octanal, and butanal, are known to impart a “grassy flavor.” The detection of fatty acid-derived aldehydes (e.g., hexanal) suggests that there are varietal or regional differences in the activity of lipid oxidation metabolic pathways [45]. Branched-chain aldehydes, exemplified by 3-methyl butanal, are produced from leucine through Strecker degradation and contribute to chocolate and malt flavors [16]. Ketones, including 2,3-pentadione and 2-pentanone, are generated as by-products of fatty acid β-oxidation.

## 4. Conclusions

Using a chemometrics-driven approach, this study established volatile fingerprints for kiwifruit variety and origin discrimination. An OPLS-DA model built from HS-GC-IMS data successfully classified samples, and analysis of VIP scores (VIP > 1) and *p*-values (*p* < 0.05) revealed distinct marker compounds: 2-vinyl-5-methylfuran, ethyl formate, and 1-penten-3-one for red-fleshed (Yunnan); limonene, methyl hexanoate, 1-propanol, and 1-hexanol for green-fleshed (Shaanxi, Yunnan); and methyl salicylate and 3-hydroxy-2-butanone for yellow-fleshed (Henan) kiwifruits. With high model robustness (Q^2^Y > 0.97) and promising test set classification, this work offers a practical, HS-GC-IMS-based framework for kiwifruit authentication. The metabolic discussions herein are literature-based hypotheses; further mechanistic insights require integrated omics and controlled environmental studies.

## Figures and Tables

**Table 1:** Sampling information of kiwifruits.

Variety	Region	Total Number	Harvest Time	Fruit Firmness
Red-fleshed (R)	Yunnan (YN, 15), Sichuan (SC, 9), Shanxi (SX, 3)	27	Early August	3.9~4.9 N/cm^2^
Green-fleshed (G)	Yunnan (YN, 12), Chongqing (CQ, 3), Shanxi (SX, 9), Hunan (HUN, 6), Guizhou (GZ, 3), Foreign Country (FC, New Zealand, 3)	36	Mid-October	5.9~6.9 N/cm^2^
Yellow-fleshed (Y)	Yunnan (YN, 9), Sichuan (SC, 9), Shanxi (SX, 3), Henan (HEN, 6), Foreign Country (FC, New Zealand, 3)	30	Early October	8.8~9.8 N/cm^2^

Notes: (1) The numbers in parentheses indicate the sampling quantities of kiwifruits from different geographical origins (for instance there were 15 samples from Yunnan). (2) Harvest Time expresses the harvest start time of different types of kiwifruits. (3) Fruit Firmness denotes the fruit firmness of different kiwifruit types at the same maturity stage. (4) Samples for each production region-variety combination were collected from representative large-scale cultivation bases in the local area. During sampling, at least 5 healthy and vigorously growing plants were randomly selected in each base, and approximately 1 kg of fruits at commercial maturity, with uniform size, free from mechanical damage, diseases and insect pests, were harvested from each plant. All fruits collected from the same base were mixed and regarded as one biological replicate. Ultimately, 3 independent biological replicates were obtained for each production region-variety combination.

## Data Availability

The original contributions presented in this study are included in the article/Supplementary Materials. Further inquiries can be directed to the corresponding authors.

## Supplementary Materials

The following supporting information can be downloaded at: https://www.mdpi.com/article/10.3390/foods15010152/s1, Table S1: Volatile components of kiwifruit; Table S2: The comparative results between model construction and cross-validation.

## References

[1] Zhong C., Huang W., Li D., Zhang Q., Li L. (2021). Analysis of the Development of the Global Kiwifruit Industry and Trends in Fresh Fruit Trade. Chin. Fruit Trees.

[2] Zhang J., Mo Z., Xuan J., Jia X., Liu Y., Guo Z. (2013). Advance of Research on Flesh Color Related Pigment Metabolism in Kiwifruit. Chin. Agric. Sci. Bull..

[3] Xu X., Zhang Q. (2003). Researches and Utilizations of Germplasm Resourceof Kiwifruit in China. Chin. Bull. Bot..

[4] Du D., Xu M., Wang J., Gu S., Zhu L., Hong X. (2019). Tracing internal quality and aroma of a red-fleshed kiwifruit during ripening by means of GC-MS and E-nose. RSC Adv..

[5] Lan T., Gao C., Yuan Q., Wang J., Zhang H., Sun X., Lei Y., Ma T. (2021). Analysis of the Aroma Chemical Composition of Commonly Planted Kiwifruit Cultivars in China. Foods.

[6] Mitalo O.W., Tokiwa S., Kondo Y., Otsuki T., Galis I., Suezawa K., Kataoka I., Doan A.T., Nakano R., Ushijima K. (2019). Low Temperature Storage Stimulates Fruit Softening and Sugar Accumulation Without Ethylene and Aroma Volatile Production in Kiwifruit. Front. Plant Sci..

[7] Tian X., Zhu L., Yang N., Song J., Zhao H., Zhang J., Ma F., Li M. (2021). Proteomics and Metabolomics Reveal the Regulatory Pathways of Ripening and Quality in Post-Harvest Kiwifruits. J. Agric. Food Chem..

[8] Ding S., Qiu M., Cao Y., Pan L. (2025). Identification of Different Volatile Components in Fresh andProcessed Atractylodis Macrocephala Rhizoma Based onGC-IMS. J. Instrum. Anal..

[9] Tian X., Li Z.J., Chao Y.Z., Wu Z.Q., Zhou M.X., Xiao S.T., Zeng J., Zhe J. (2020). Evaluation by electronic tongue and headspace-GC-IMS analyses of the flavor compounds in dry-cured pork with different salt content. Food Res. Int..

[10] Wang S., Chen H., Sun B. (2020). Recent progress in food flavor analysis using gas chromatography–ion mobility spectrometry (GC–IMS). Food Chem..

[11] Gao L., Zhang L., Liu J., Zhang X., Lu Y. (2023). Analysis of the Volatile Flavor Compounds of Pomegranate Seeds at Different Processing Temperatures by GC-IMS. Molecules.

[12] Sun P., Xu B., Wang Y., Lin X., Chen C., Zhu J., Jia H., Wang X., Shen J., Feng T. (2022). Characterization of volatile constituents and odorous compounds in peach (*Prunus persica* L) fruits of different varieties by gas chromatography–ion mobility spectrometry, gas chromatography–mass spectrometry, and relative odor activity value. Front. Nutr..

[13] Man L., Ren W., Sun M., Du Y., Chen H., Qin H., Chai W., Zhu M., Liu G., Wang C. (2023). Characterization of donkey-meat flavor profiles by GC–IMS and multivariate analysis. Front. Nutr..

[14] Huang H. (2025). Study on Aroma Differences of Tea Leaves with Different Fermentation Types and Storage Times Based on Electronic Nose, HS-GC-IMS and HS-SPME-GC-MS. Master’s Thesis.

[15] Ye Y., Li K., Bi Y., Lin T., Chen D., Du L. (2025). Comprehensive evaluation of Actinidia chinensis Planch quality in major producing areas of China based on entropy weight-technique for order preference by similarity to ideal solution method. J. Food Saf. Qual..

[16] Zhao H., Zhang S., Ma D., Liu Z., Qi P., Wang Z., Di S., Wang X. (2024). Review of fruits flavor deterioration in postharvest storage: Odorants, formation mechanism and quality control. Food Res. Int..

[17] Xi W., Zhang L., Liu S., Zhao G. (2020). The Genes of CYP, ZEP, and CCD1/4 Play an Important Role in Controlling Carotenoid and Aroma Volatile Apocarotenoid Accumulation of Apricot Fruit. Front. Plant Sci..

[18] Zamljen T., Grohar M.C., Medic A. (2024). Mint-Scented Species in Lamiaceae: An Abundant and Varied Reservoir of Phenolic and Volatile Compounds. Foods.

[19] Rey-Serra P., Mnejja M., Monfort A. (2022). Inheritance of esters and other volatile compounds responsible for the fruity aroma in strawberry. Front. Plant Sci..

[20] Melini F., Melini V. (2024). Role of Microbial Fermentation in the Bio-Production of Food Aroma Compounds from Vegetable Waste. Fermentation.

[21] Lipinski S., Lindekamp N., Funck N., Cramer B., Humpf H.-U. (2023). Determination of furan and alkylfuran in breakfast cereals from the European market and their correlation with acrylamide levels. Eur. Food Res. Technol..

[22] Mall V., Sellami I., Schieberle P. (2018). New Degradation Pathways of the Key Aroma Compound 1-Penten-3-one during Storage of Not-from-Concentrate Orange Juice. J. Agric. Food Chem..

[23] Kumari S., Pande K.K., Movi S., Kumar A., Solanki P., Rajput R., Das J., Das R. (2024). Climate Change Impact on Fruit Crops and Mitigation through Climate-Smart Production Practices. J. Sci. Res. Rep..

[24] Wang L., Tang P., Zhang P., Lu J., Chen Y., Xiao D., Guo X. (2024). Unraveling the aroma profiling of Baijiu: Sensory characteristics of aroma compounds, analytical approaches, key odor-active compounds in different Baijiu, and their synthesis mechanisms. Trends Food Sci. Technol..

[25] Guan S., Yang F., Yao J., Liu C., Wang R., Ruan M., Yao Z., Liu C., Wan H., Li Z. (2025). Dynamic changes in volatile organic compounds of cherry tomato fruits during storage at different temperatures using HS-GC-IMS. Food Res. Int..

[26] Wang C., Xing J., Chin C.-K., Ho C.-T., Martin C.E. (2001). Modification of fatty acids changes the flavor volatiles in tomato leaves. Phytochemistry.

[27] Supriyadi, Suzuki M., Wu S., Tomita N., Fujita A., Watanabe N. (2003). Biogenesis of Volatile Methyl Esters in Snake Fruit (*Salacca edulis*, Reinw) cv. Pondoh. Biosci. Biotechnol. Biochem..

[28] Zhang A., Zhang Q., Li J., Gong H., Fan X., Yang Y., Liu X., Yin X. (2020). Transcriptome co-expression network analysis identifies key genes and regulators of ripening kiwifruit ester biosynthesis. BMC Plant Biol..

[29] Zhang B., Yin X.-R., Li X., Yang S.-L., Ferguson I.B., Chen K.-S. (2009). Lipoxygenase Gene Expression in Ripening Kiwifruit in Relation to Ethylene and Aroma Production. J. Agric. Food Chem..

[30] Qian X., Xu X.-Q., Yu K.-J., Zhu B.-Q., Lan Y.-B., Duan C.-Q., Pan Q.-H. (2016). Varietal Dependence of GLVs Accumulation and LOX-HPL Pathway Gene Expression in Four Vitis vinifera Wine Grapes. Int. J. Mol. Sci..

[31] Fang X., Xu W., Jiang G., Sui M., Xiao J., Ning Y., Niaz R., Wu D., Feng X., Chen J. (2024). Monitoring the dynamic changes in aroma during the whole processing of Qingzhuan tea at an industrial scale: From fresh leaves to finished tea. Food Chem..

[32] Api A.M., Bartlett A., Belsito D., Botelho D., Bruze M., Bryant-Freidrich A., Burton G.A., Cancellieri M.A., Chon H., Dagli M.L. (2024). RIFM fragrance ingredient safety assessment, benzoic acid, 2-hydroxy-5-methyl-, methyl ester, CAS Registry Number 22717-57-3. Food Chem. Toxicol..

[33] Tian H., Yu B., Yu H., Chen C. (2020). Evaluation of the synergistic olfactory effects of diacetyl, acetaldehyde, and acetoin in a yogurt matrix using odor threshold, aroma intensity, and electronic nose analyses. J. Dairy Sci..

[34] Liu X., Feng Y., Li S., Li D., Yu J., Zhao Z. (2023). Jasmonate-induced MdMYC2 improves fruit aroma during storage of ‘Ruixue’ apple based on transcriptomic, metabolic and functional analyses. LWT.

[35] Pott D.M., Durán-Soria S., Allwood J.W., Pont S., Gordon S.L., Jennings N., Austin C., Stewart D., Brennan R.M., Masny A. (2023). Dissecting the impact of environment, season and genotype on blackcurrant fruit quality traits. Food Chem..

[36] Imahori Y., Yamamoto K., Tanaka H., Bai J. (2013). Residual effects of low oxygen storage of mature green fruit on ripening processes and ester biosynthesis during ripening in bananas. Postharvest Biol. Technol..

[37] Chen S., Zhang F., Ananta E., Muller J.A., Liang Y., Lee Y.K., Liu S.-Q. (2024). Co-Inoculation of Latilactobacillus sakei with Pichia kluyveri or Saccharomyces boulardii Improves Flavour Compound Profiles of Salt-Free Fermented Wheat Gluten. Fermentation.

[38] Liu W., Fan M., Sun S., Li H. (2020). Effect of mixed fermentation by Torulaspora delbrueckii, Saccharomyces cerevisiae, and Lactobacillus plantarum on the sensory quality of black raspberry wines. Eur. Food Res. Technol..

[39] Liu Z., Cai S., Zhang S., Xiao Y., Devahastin S., Guo C., Wang Y., Wang T., Yi J. (2023). A systematic review on fermented chili pepper products: Sensorial quality, health benefits, fermentation microbiomes, and metabolic pathways. Trends Food Sci. Technol..

[40] Varela C., Torrea D., Schmidt S.A., Ancin-Azpilicueta C., Henschke P.A. (2012). Effect of oxygen and lipid supplementation on the volatile composition of chemically defined medium and Chardonnay wine fermented with Saccharomyces cerevisiae. Food Chem..

[41] Kracht O.N., Ammann A.-C., Stockmann J., Wibberg D., Kalinowski J., Piotrowski M., Kerr R., Brück T., Kourist R. (2017). Transcriptome profiling of the Australian arid-land plant Eremophila serrulata (A.DC.) Druce (Scrophulariaceae) for the identification of monoterpene synthases. Phytochemistry.

[42] Makangara J.J., Mshandete A.M., Mbega E.R., Nyika J.R., Mbago F., Ndilanha E.G., Nyika R.J., Nyika J.J. (2024). Review on the secondary metabolites, biological properties, and ethnomedicinal uses of the component species of the buheri wa afya formula used to treat COVID-19 in Tanzania. Phytomedicine Plus.

[43] Wang B., Wu W., Liu J., Soladoye O.P., Ho C.-T., Zhang Y., Fu Y. (2023). Flavor mystery of spicy hot pot base: Chemical understanding of pungent, numbing, umami and fragrant characteristics. Trends Food Sci. Technol..

[44] Valentoni A., Melis R., Sanna M., Porcu M.C., Rodolfi M., Braca A., Bianco A., Zara G., Budroni M., Anedda R. (2023). Fruit Beer with the Bisucciu Sardinian Apricot Cultivar (*Prunus armeniaca* L.): A Technological and Analytical Approach. Fermentation.

[45] Zhang H., Zhu X., Xu R., Yuan Y., Abugu M.N., Yan C., Tieman D., Li X. (2023). Postharvest chilling diminishes melon flavor via effects on volatile acetate ester biosynthesis. Front. Plant Sci..

