# Supplementary material for: Characterization of Volatile Profile of Different Kiwifruits (Actinidia chinensis Planch) Varieties and Regions by Headspace-Gas Chromatography-Ion Mobility Spectrometry
Source: Foods. 2026 Jan 3;15(1):152. doi: 10.3390/foods15010152 (PMC12786179; doi:10.3390/foods15010152)
Supplement: Supplementary file 1 [file foods-15-00152-s001.zip › Table S1. Volatile components of kiwifruit.pdf]

Table S1. Volatile components of kiwifruit

No.	Name	CAS#	Formula	MW	RI	Rt [sec]	Dt [a.u.]	Type	Oder Characteristics
F1	(E)-3-Undecene_M	C1002682	$C_{11}H_{22}$	154.3	1144.5	495.854	1.5400	hydrocarbon	
F2	(E)-3-Undecene_D	C1002682	$C_{11}H_{22}$	154.3	1146.3	499.018	1.6834	hydrocarbon	
F3	Anisole	C100663	C_7H_8O	108.1	1314.5	788.581	1.2446	ether	anise odor
F4	Benzoic acid, 2-hydroxy-, methyl ester	C119368	$C_8H_8O_3$	152.1	1289.5	744.964	1.2171	ester	wintergreen
F5	Geraniol	C106241	$C_{10}H_{18}O$	154.3	1233.8	650.003	1.6896	alcohol	floral, fruity, waxy
F6	1,3,5-Trimethylbenzene	C108678	C_9H_{12}	120.2	1244.3	666.879	1.0458	hydrocarbon	
F7	2-Ethylhexyl vinyl ether_M	C103446	$C_{10}H_{20}O$	156.3	1167.5	538.004	1.3355	ether	
F8	Ethyl 2-butenoate	C10544635	$C_6H_{10}O_2$	114.1	1175.6	553.697	1.1901	ester	fruity
F9	2-Vinyl-5-methylfuran	C10504139	C_7H_8O	108.1	1147.3	500.879	1.2756	furan	fruity, sweet, nutty
F10	4-Vinylcyclohexene	C100403	C_8H_{12}	108.2	1041.0	355.769	1.4351	hydrocarbon	
F11	Ethyl propanoate-D	C105373	$C_5H_{10}O_2$	102.1	968.60	292.897	1.4504	ester	sweet fruity, rum juicy
F12	(E)-4-Nonene	C10405853	C_9H_{18}	126.2	973.50	296.230	1.5040	hydrocarbon	gasoline-like
F13	1-Pentanal	C110623	$C_5H_{10}O$	86.10	1002.8	318.174	1.4283	aldehyde	bready, fruity, nutty
F14	1-Octanal	C124130	$C_8H_{16}O$	128.2	1299.7	763.058	1.4134	aldehyde	fatty, fruity
F15	(E)-2-Heptenal_M	C18829555	$C_7H_{12}O$	112.2	1331.8	819.470	1.2599	aldehyde	green, vegetable, fatty
F16	(E)-2-Heptenal_D	C18829555	$C_7H_{12}O$	112.2	1331.9	819.710	1.6697	aldehyde	green, vegetable, fatty
F17	Ethyl caproate_D	C123660	$C_8H_{16}O_2$	144.2	1251.7	679.107	1.7952	ester	sweet, fruity, waxy, green
F18	Ethyl caproate_M	C123660	$C_8H_{16}O_2$	144.2	1251.8	679.358	1.3424	ester	sweet, fruity, waxy, green
F19	(E)-2-Hexen-1-al_D	C6728263	$C_6H_{10}O$	98.10	1219.9	628.288	1.5101	aldehyde	green, fatty, cheesy
F20	(E)-2-Hexen-1-al_M	C6728263	$C_6H_{10}O$	98.10	1220.3	628.896	1.1808	aldehyde	green, fatty, cheesy
F21	Heptaldehyde	C111717	$C_7H_{14}O$	114.2	1196.0	592.583	1.3409	aldehyde	green, vegetable
F22	(E)-2-Pentenal_M	C1576870	C_5H_8O	84.10	1144.1	495.244	1.1026	aldehyde	green, fruity, vegetable
F23	(E)-2-Pentenal_D	C1576870	C_5H_8O	84.10	1144.2	495.288	1.3556	aldehyde	green, fruity, vegetable
F24	α-Pinene	C80568	$C_{10}H_{16}$	136.2	1023.9	338.400	1.2051	alkene	
F25	2-Pentyl furan	C3777693	$C_9H_{14}O$	138.2	1223.7	634.122	1.2464	furan	fruity, green, earthy, beany, vegetable, metallic

F26	3-Methyl butanal	C590863	$C_5H_{10}O$	86.10	925.20	265.166	1.4031	aldehyde	chocolate, fruity, fatty
F27	2-Methyl-1-propyl acetate_D	C110190	$C_6H_{12}O_2$	116.2	1024.5	339.083	1.6141	ester	fruity
F28	2-Methyl-1-propyl acetate_M	C110190	$C_6H_{12}O_2$	116.2	1024.7	339.220	1.2348	ester	fruity
F29	1- Butanol_M	C71363	$C_4H_{10}O$	74.10	1153.2	511.470	1.1834	alcohol	alcohol-like
F30	1- Butanol_D	C71363	$C_4H_{10}O$	74.10	1153.1	511.302	1.3885	alcohol	alcohol-like
F31	Acetic acid ethyl ester_D	C141786	$C_4H_8O_2$	88.10	903.00	251.984	1.3365	ester	fruity
F32	Acetic acid ethyl ester_M	C141786	$C_4H_8O_2$	88.10	905.70	253.533	1.1085	ester	fruity
F33	Butanal	C123728	C_4H_8O	72.10	830.20	213.207	1.2774	aldehyde	chocolate
F34	Ethyl formate	C109944	$C_3H_6O_2$	74.10	818.80	207.698	1.0727	ester	pleasant aromatic
F35	Methyl acetate_M	C79209	$C_3H_6O_2$	74.10	847.80	221.986	1.0293	ester	sweet
F36	Methyl acetate_D	C79209	$C_3H_6O_2$	74.10	846.90	221.543	1.1940	ester	sweet
F37	Propanal	C123386	C_3H_6O	58.10	818.70	207.655	1.1440	aldehyde	earthy, whiskey, chocolate, nutty
F38	2-Ethyl furan_M	C3208160	C_6H_8O	96.10	960.70	287.668	1.0442	furan	beany, chocolate, bready, malty, coffee, nutty
F39	2-Ethyl furan_D	C3208160	C_6H_8O	96.10	976.20	298.054	1.3085	furan	beany, chocolate, bready, malty, coffee, nutty
F40	4-Methyl-2-pentanone	C108101	$C_6H_{12}O$	100.2	1022.5	337.042	1.4817	ketone	green
F41	Butanoic acid ethyl ester	C105544	$C_6H_{12}O_2$	116.2	1050.7	365.989	1.5548	ester	fruity
F42	2-Propanone	C67641	C_3H_6O	58.10	835.20	215.653	1.1131	ketone	pungent, sweet
F43	(+)-Limonene	C138863	$C_{10}H_{16}$	136.2	1198.8	596.686	1.2881	terpenoid	fruity
F44	(Z)-Ocimene	C470826	$C_{10}H_{18}O$	154.3	1217.0	623.848	1.2969	terpenoid	floral, herb, sweet
F45	1-Hexanal_D	C66251	$C_6H_{12}O$	100.2	1072.9	390.565	1.5621	aldehyde	green
F46	1-Hexanal_M	C66251	$C_6H_{12}O$	100.2	1072.4	390.013	1.2149	aldehyde	green
F47	2-Butanone, 3-hydroxy	C513860	$C_4H_8O_2$	88.10	1289.1	744.319	1.3073	ketone	creamy, fatty, buttery
F48	2-Hexanone	C591786	$C_6H_{12}O$	100.2	1121.9	457.714	1.1858	ketone	fruity, fungal, meaty, buttery
F49	2,3-Pentadione	C600146	$C_5H_8O_2$	100.1	1062.9	379.330	1.2209	ketone	sweet, butter, creamy, caramel, nutty, cheese
F50	2-Pentanone_M	C107879	$C_5H_{10}O$	86.10	1012.0	326.851	1.1350	ketone	sweet, fruity, wine, woody
F51	2-Pentanone_D	C107879	$C_5H_{10}O$	86.10	1010.0	324.961	1.3723	ketone	sweet, fruity, wine, woody
F52	1-Penten-3-one_D	C1629589	C_5H_8O	84.10	1037.8	352.441	1.3128	ketone	peppery, mustard, garlic, onion

F53	1-Penten-3-one_M	C1629589	C_5H_8O	84.10	1037.0	351.680	1.0785	ketone	peppery, mustard, garlic, onion
F54	2-Methyl propanal	C78842	C_4H_8O	72.10	793.80	196.107	1.0889	aldehyde	pungent
F55	1-Propanol	C71238	C_3H_8O	60.10	1062.5	378.859	1.2522	alcohol	alcohol-like
F56	1-Pentanol	C71410	$C_5H_{12}O$	88.10	1236.5	654.417	1.5170	alcohol	alcohol-like
F57	1 -Hexanol_M	C111273	$C_6H_{14}O$	102.2	1367.5	887.248	1.3307	alcohol	sweet, mild
F58	1 -Hexanol_D	C111273	$C_6H_{14}O$	102.2	1367.3	886.706	1.6517	alcohol	sweet
F59	Butyl propanoate	C590012	$C_7H_{14}O_2$	130.2	1131.9	474.298	1.2710	ester	earthy, sweet, floral
F60	Butanoic acid, propyl ester	C105668	$C_7H_{14}O_2$	130.2	1134.1	477.988	1.6879	ester	
F61	Hexanoic acid, methyl ester	C106707	$C_7H_{14}O_2$	130.2	1197.8	595.187	1.6744	ester	fruity, bacon
F62	Butanoic acid, methyl ester	C623427	$C_5H_{10}O_2$	102.1	986.10	304.928	1.1385	ester	fruity
F63	2-Ethylhexyl vinyl ether_D	C103446	$C_{10}H_{20}O$	156.3	1167.2	537.365	1.8049	ether	
F64	3-Methylfuran	C930278	C_5H_6O	82.10	841.10	218.597	1.1535	furan	
F65	Methyl pentanoate	C624248	$C_6H_{12}O_2$	116.2	1101.2	425.265	1.5598	ester	sweet, green, fruity, nutty
F66	Butanol	C71363	$C_4H_{10}O$	74.10	1123.4	460.139	1.3635	alcohol	camphor-like
F67	3-Pentanol	C584021	$C_5H_{12}O$	88.10	1121.1	456.453	1.5258	alcohol	sweet, herbal, oily, nutty
F68	β-Pinene	C127913	$C_{10}H_{16}$	136.2	1117.7	450.925	1.2103	terpenoid	woody, green
F69	1-Nonanal	C124196	$C_9H_{18}O$	142.2	1401.9	957.646	1.4301	aldehyde	fatty-floral, waxy
F70	Benzaldehyde	C100527	C_7H_6O	106.1	1539.9	1301.56	1.1629	aldehyde	fruity
F71	α-Terpinene	C99865	$C_{10}H_{16}$	136.2	1186.8	576.005	1.2128	terpenoid	
F72	Ethyl propanoate-M	C105373	$C_5H_{10}O_2$	102.1	944.20	276.991	1.1640	ester	sweet fruity, rum juicy
F73	2-Methylbutanoic acid, methyl ester	C868575	$C_6H_{12}O_2$	116.2	1008.7	323.771	1.1960	ester	fruity
F74	Acetic acid butyl ester	C123864	$C_6H_{12}O_2$	116.2	1086.2	405.994	1.6171	ester	fruity
F75	Ethyl 2-methy lpropionate	C97621	$C_6H_{12}O_2$	116.2	976.30	298.131	1.5523	ester	sweet, fruity, alcoholic-like
F76	1-Hexen-3-one	C1629603	$C_6H_{10}O$	98.10	1095.2	416.814	1.2760	ketone	rubber-like, pungent
F77	Methyl (E)-2-butenoate	C623438	$C_5H_8O_2$	100.1	1094.5	416.002	1.3698	ester	green, fruity

Source: (https://www.chemicalbook.com/ProductIndex.aspx) (https://www.vcf-online.nl/VcfHome.cfm).
