# Supplementary material for: Characterization of Volatile Profile of Different Kiwifruits (Actinidia chinensis Planch) Varieties and Regions by Headspace-Gas Chromatography-Ion Mobility Spectrometry
Source: Foods. 2026 Jan 3;15(1):152. doi: 10.3390/foods15010152 (PMC12786179; doi:10.3390/foods15010152)
Supplement: Supplementary file 1 [file foods-15-00152-s001.zip › Table S2. The comparative results between model construction and cross-validation.pdf]

Table S2. The comparative results between model construction and cross-validation

Type	RMSECV	RMSEE	Class (Origin)	Sensitivity (%)	Specificity (%)	Precision (%)	F1-Score
Red-fleshed	0.0593211	0.0434695	YN	100	100	100	1
			SC	100	100	100	1
			SX	100	100	100	1
			prediction accuracy (%)		100		
Green-fleshed	0.070779	0.0503641	YN	100	100	100	1
			CQ	100	100	100	1
			SX	100	100	100	1
			HUN	100	100	100	1
			GZ	100	100	100	1
			FC	100	100	100	1
			prediction accuracy (%)		100		
Yellow-fleshed	0.0417436	0.0338597	YN	100	100	100	1
			SC	100	100	100	1
			SX	100	100	100	1
			HEN	100	100	100	1
			FC	100	100	100	1
			prediction accuracy (%)		100		
